# Supplementary material for: Ensemble-averaged Rabi oscillations in a ferromagnetic CoFeB film
Source: Nat Commun. 2017 Jun 28;8:16004. doi: 10.1038/ncomms16004 (PMC5493749; doi:10.1038/ncomms16004)
Supplement: Supplementary Information [file ncomms16004-s1.pdf]

Type of file: PDF
Size of file: 0 KB
Title of file for HTML: Supplementary Information
Description: Supplementary Figures, Supplementary Notes and Supplementary References

Type of file: PDF
Size of file: 0 KB
Title of file for HTML: Peer Review File
Description:

Supplementary Note 1. Relaxation in ferromagnets

The T_1 and T_2^* relaxation times defined in the Bloch-Bloembergen representation[1,2] describe the spin-lattice and the transverse relaxation times, respectively, with the latter being responsible also for the inhomogeneities, as defined by:

$$\begin{aligned} \frac{dM_{x,y}}{dt} &= \gamma\left(\mathbf{M}\times\mathbf{H}\right)_{x,y} - \frac{M_{x,y}}{T_2^*} \\ \frac{dM_z}{dt} &= \gamma\left(\mathbf{M}\times\mathbf{H}\right)_z - \frac{M_z - M_0}{T_1} \end{aligned} \qquad (1)$$

for which M_i is the component of the magnetization along the i coordinate with **M** being the magnetization vector, **H** being the magnetic field vector, and M_0 is the steady state magnetization along the z axis.

Alternatively, but not equally, the relaxation processes can be described using the Gilbert damping coefficient, α, which takes into account the spin-lattice and transverse decays simultaneously, as used in the Landau-Lifshitz-Gilbert equation:

$$\frac{d\mathbf{M}}{dt} = -\gamma\mathbf{M}\times\mathbf{H} + \alpha\frac{1}{M_s}\mathbf{M}\times\frac{d\mathbf{M}}{dt}, \qquad (2)$$

while the inhomogeneous broadening is modeled through the variation in H_{Keff}, ΔH_{Keff}, as in the works by Shaw et al.[3] and by Iihama et al.,[4] for example.

T_1, T_2^*, and the Gilbert damping relaxations can be represented in a graphical manner as shown in Supplementary Fig. 1. Namely, α is a nontrivial, time dependent, combination of T_1 and T_2^*.

Supplementary Note 2. Numerical simulation

Calculation of the non-adiabatic interaction was carried out by numerically integrating the Landau-Lifshitz-Gilbert equation. Since the calculation does not account for the inhomogeneous broadening, it describes the experiment in a qualitative manner. In the calculation, the steady precessional state was first obtained before applying the perturbation. Two sources for the perturbation were introduced that gave the best results: quenching of the magnetization and introduction of a momentary phenomenological magnetic field. The latter was required in order to reproduce the phase response at positive times near $t = 0$, namely the curvature in the vertical contours of Supplementary Fig. 2 appearing at times that immediately follow the pump. In Supplementary Fig. 2, the wave fronts shift to later times as the field increases to a value of ~ 440 mT. When the field is further increased, the wave fronts shift to earlier times as in the experiment. Since the magnetization acquires a phase shift that is associated with the resonance at a field of about 450 mT, presenting an additional phenomenological magnetic field causes the magnetization to alter its motion. This additional torque was applied in the form of a 3 ps pulsed magnetic field of 60 mT which lied in the film plane orthogonal to the axis of

precession. The recovery profile of the magnetization after quenching consisted of two time constants of 50 ps and 500 ps while the modulation depth was 5%.

The simulation result is shown in Supplementary Fig. 3. Imprints of Rabi nutations on the amplitude of the precessions are readily seen. The formation of the valley as in Fig. 1F of main text is also observed. This valley however appears also at field values which are larger than the resonance field in contrast to the measurement. At magnetic fields near resonance and immediately after zero time, similar contours to the ones shown in Supplementary Fig. 2 are seen.

Supplementary Note 3. Interaction of optical pulses with the magnetic media

The optical pump pulses in this work are responsible for initiating the nonadiabatic regime by perturbing the ferromagnetic order after the driven steady state precessions are reached. The discussion regarding the excitation of the ferromagnetic order by ultrashort optical pulses was largely stimulated by the pioneering work of Beaurepaire et al.[5] In that work, the time-resolved MOKE method was used to study the light-ferromagnet interaction and since then the topic has been subject to much discussion and debate. Beaurepaire et al. attributed the interaction to thermal effects where electrons are optically excited into hot high energetic states after which they lose their energy to the lattice on timescales of ~ 1 ps. Consequently, the magnetization state is altered by the Curie-Weiss law. Later models included the dissipation of angular momentum by Elliott-Yafet type scattering into the lattice[6] and were shown to agree with the experimental observations.[7,8]

Our numerical simulations suggest that the thermal effects cannot account on their own for the measured results. In order to reproduce the measured field dependent phase responses near $t = 0$ (Supplementary Fig. 2), an effective torque of nonthermal origin was introduced in the form of a 3 ps pulsed magnetic field of 60 mT which lied along the $\hat{y}$ direction. Despite the better agreement with the experiment, the uniqueness or origin of this nonthermal field cannot be unequivocally verified.

Nonthermal effects which result in generation of effective fields can be classified as optomagnetic effects that do not require the absorption of electromagnetic radiation or as photomagnetic effects that involve the absorption of light.[9]

In the study by Hansteen et al.[10], photo induced nonthermal effects were thoroughly addressed in ferrimagnetic garnet films in which linearly polarized optical pulses, as the ones used in our experiment, were shown to be responsible for a long-lived modification of the anisotropy field. These experiments were well described by optical rectification as well as by a linear magneto-electric effect which depended on the symmetry of the sample. The interaction with the light was attributed to transfer of charge between Fe^{4+} and Fe^{3+} ions thereby effectively displacing the ions to sites of different symmetry and inducing a change in the anisotropy field.

The non-absorptive inverse Faraday effect[10-13], belonging to the family of optomagnetic effects, may account for the transient field as well although it is responsible for inducing a field along the optical propagation axis and that exists as long as the optical pulse is present. This mechanism involves a stimulated Raman-like coherent optical scattering that results in high speed and efficiency of the excitation[10]. The short duration of the optical pulses allows for two frequency components within its their wide

spectrum to take part in the stimulated coherent Raman process that in the presence of strong spin-orbit coupling results in larger spin-flip probabilities.

Recently Khorsand et al.[14] discussed the role of magnetic circular dichroism (MCD) for which a difference in energy absorption depending on the polarization of light exists. Their conclusion was reached by analyzing all-optical helicity dependent switching experiments in ferrimagnetic GdFeCo based films, specifically, from the observed correlations between the switching optical intensity window and the MCD process. A closely related hybrid photo-thermal magnetic interaction was also discussed[15], recently as well, where a thermal excitation over the anisotropy energy barrier together with MCD create a net magnetization over a sequence of laser pulses.

Other possible sources that may induce a transient field include demagnetization and anisotropy change induced field[16,17], plasma effects induced by the optical pump following spin-flip scattering as well as spin wave emission at the laser spot.[18]

More detailed discussions of the topic can be found in the reviews by Kirilyuk[9] et al. and by Zhang et al.[19]

Supplementary Note 4. Extended coherence at high applied field and 10 GHz

Signatures of the extended coherences were even found in the phase responses at 10 GHz (Fig. 2A of main text) where τ_{eff} and τ_{int} are shorter. At negative times, before the pump pulse arrives, the phase in Fig. 2B of main text shifts slightly to negative values as H_0 increases to $\mu_0 H_0$ ~ 450 mT. This response differs from the responses measured when the optical pump is completely turned off (Supplementary Fig. 4), indicating the slight remnant coherence from the previous cycle. Moreover, the negative phase shift resembles the behavior of the phases seen immediately after $t = 0$ (Supplementary Fig. 2) implying a link between the responses manifested by long lasting coherence despite the short τ_{eff} and τ_{int} [20]. A non-coherent process, such as a thermal process would have had an equal effect for all H_0 and would not have affected the phase in the manner observed. Once more, the corresponding TR-MOKE traces show no sign of the coherent interaction after T_R, demonstrating the ability to observe details that were obscured by the ensemble dephasing.

Supplementary Figures

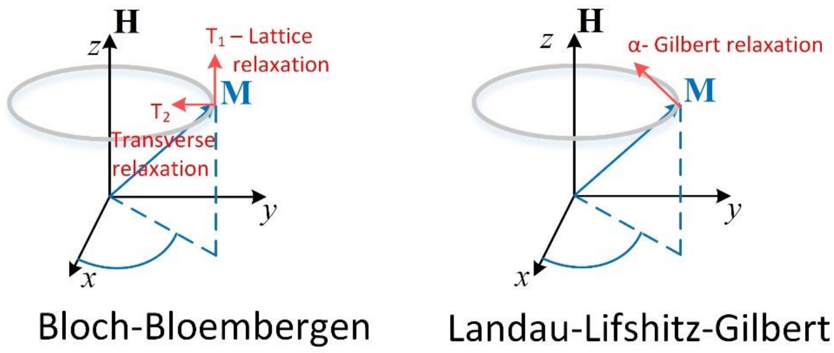

Supplementary Fig. 1. Schematic representation of dynamical equations.
Geometrical representation of the relaxation torques in the Bloch-Bloembergen and Landau-Lifshitz-Gilbert formalisms.

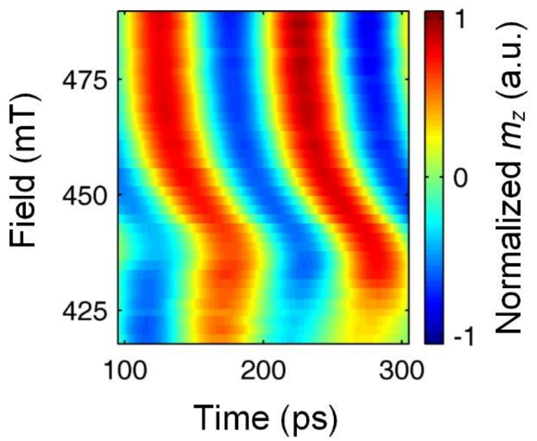

Supplementary Fig. 2. Phase response near t=0.

Close-up of the experimental results of Fig. 2A of main text for times between for times between 100 ps and 300 ps. A negative phase shift is seen as the field increases to a value of 440 mT after which a positive phase shift occurs when the field is further increased. Thermal models were not sufficient to reproduce the observed dependence of the phase on the externally applied field. Hence, a momentary pulse of magnetic field was added to the simulations which better reproduced the observed phase responses.

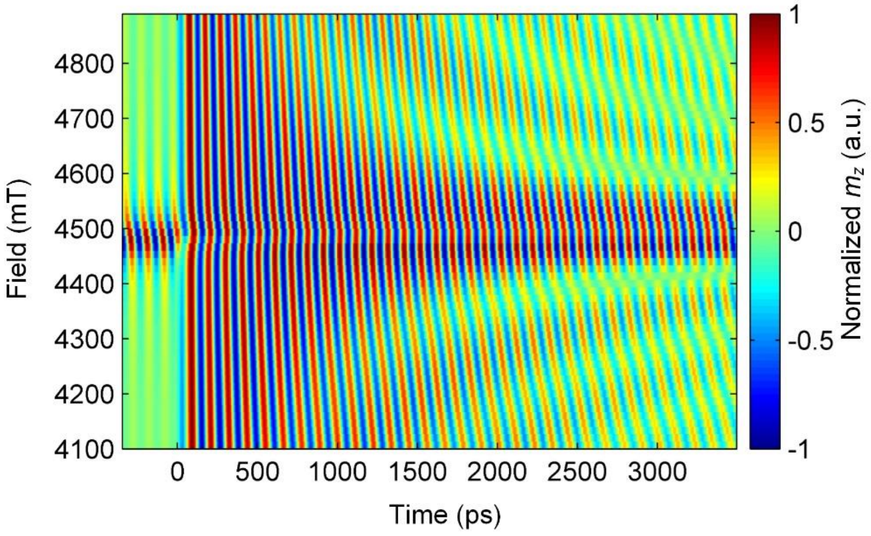

Supplementary Fig. 3. Numerical simulation result.

Calculation of the out-of-plane component of the magnetization, m_z. The response at each bias field was normalized independently to reach a maximum value of unity.

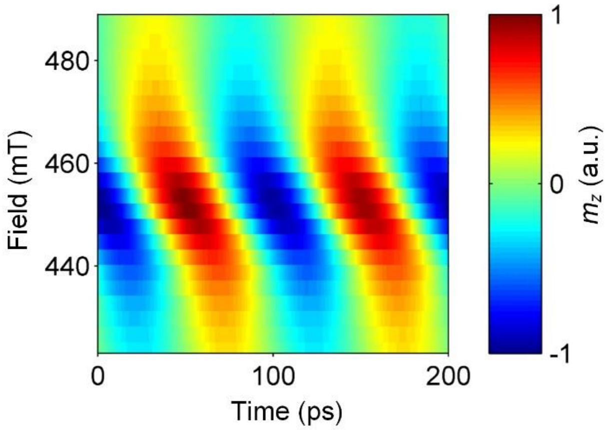

Supplementary Fig. 4. Measured response without optical pump.

Resonance response without the optical pump for the CoFeB sample. Measurement shows the out-of-plane component of the magnetization, m_z, at 10 GHz. The phase increases monotonically with the field in contrast to Fig. 2B of the main text. Data are not normalized.

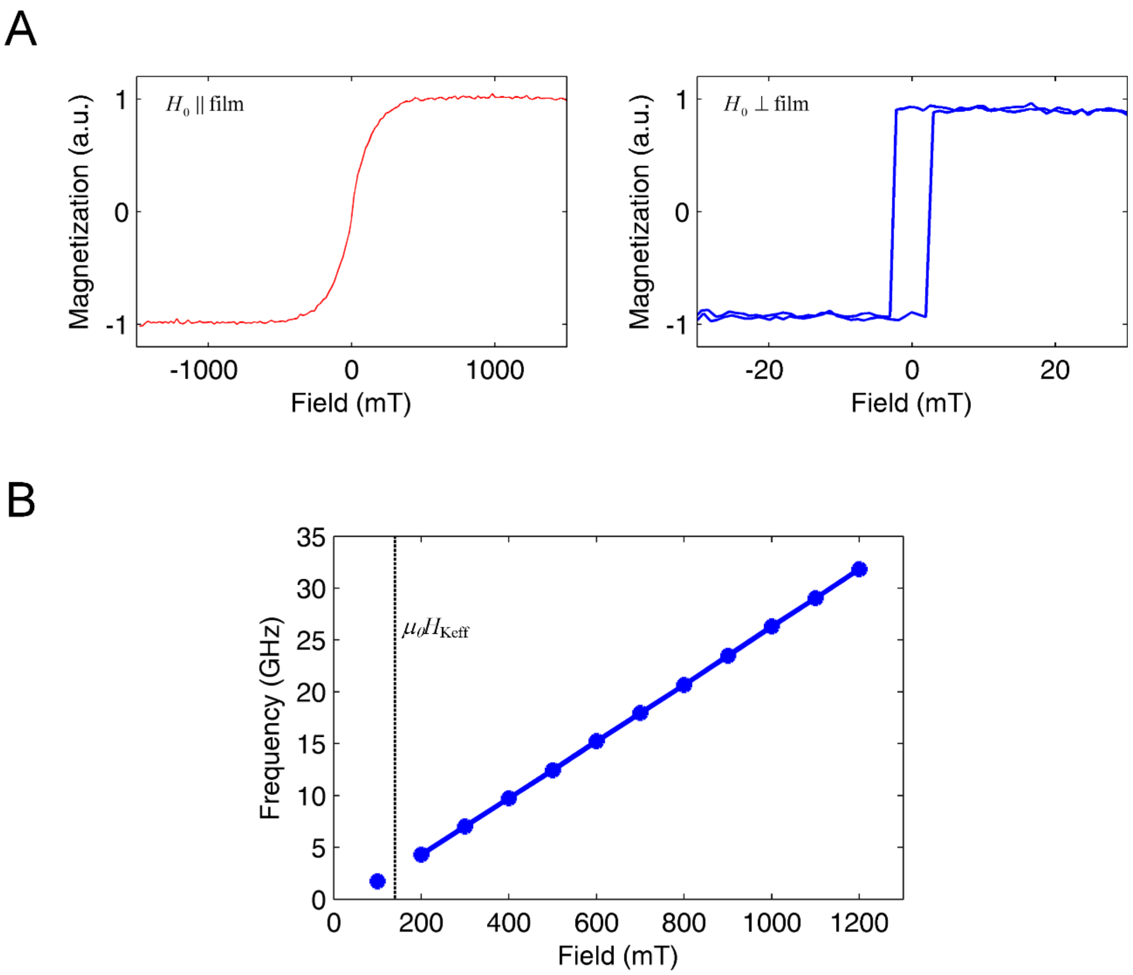

Supplementary Fig. 5. CoFeB film characteristics.

CoFeB film characterization. (A) In-plane and out-of-plane magnetization loops. (B) Frequency vs. applied field as measured in a TR-MOKE experiment. The magnetic field was applied at an angle of 4° away from the sample plane. Vertical black dashed line indicates the field value of H_{Keff}.

Supplementary references

1 Bloch, F. Nuclear Induction. *Phys. Rev.* **70**, 460-474, (1946).

2 Bloembergen, N., Purcell, E. M. & Pound, R. V. Relaxation Effects in Nuclear Magnetic Resonance Absorption. *Phys. Rev.* **73**, 679-712, (1948).

3 Shaw, J. M., Nembach, H. T. & Silva, T. J. Resolving the controversy of a possible relationship between perpendicular magnetic anisotropy and the magnetic damping parameter. *Appl. Phys. Lett.* **105**, 062406, (2014).

4 Iihama, S. *et al.* Gilbert damping constants of Ta/CoFeB/MgO(Ta) thin films measured by optical detection of precessional magnetization dynamics. *Phys. Rev. B* **89**, 174416, (2014).

5 Beaurepaire, E., Merle, J. C., Daunois, A. & Bigot, J. Y. Ultrafast Spin Dynamics in Ferromagnetic Nickel. *Phys. Rev. Lett.* **76**, 4250-4253, (1996).

6 Koopmans, B., Ruigrok, J. J. M., Longa, F. D. & de Jonge, W. J. M. Unifying Ultrafast Magnetization Dynamics. *Phys. Rev. Lett.* **95**, 267207, (2005).

7 Stamm, C. *et al.* Femtosecond modification of electron localization and transfer of angular momentum in nickel. *Nat. Mater.* **6**, 740-743, (2007).

8 Koopmans, B. *et al.* Explaining the paradoxical diversity of ultrafast laser-induced demagnetization. *Nat. Mater.* **9**, 259-265, (2010).

9 Kirilyuk, A., Kimel, A. V. & Rasing, T. Ultrafast optical manipulation of magnetic order. *Rev. Mod. Phys.* **82**, 2731-2784, (2010).

10 Hansteen, F., Kimel, A., Kirilyuk, A. & Rasing, T. Nonthermal ultrafast optical control of the magnetization in garnet films. *Phys. Rev. B* **73**, 014421, (2006).

11 Kimel, A. V. *et al.* Ultrafast non-thermal control of magnetization by instantaneous photomagnetic pulses. *Nature* **435**, 655-657, (2005).

12 Stanciu, C. D. *et al.* All-Optical Magnetic Recording with Circularly Polarized Light. *Phys. Rev. Lett.* **99**, 047601, (2007).

13 Cornelissen, T. D., Córdoba, R. & Koopmans, B. Microscopic model for all optical switching in ferromagnets. *Appl. Phys. Lett.* **108**, 142405, (2016).

14 Khorsand, A. R. *et al.* Role of Magnetic Circular Dichroism in All-Optical Magnetic Recording. *Phys. Rev. Lett.* **108**, 127205, (2012).

15 Ellis, M. O. A., Fullerton, E. E. & Chantrell, R. W. All-optical switching in granular ferromagnets caused by magnetic circular dichroism. *Sci. Rep.* **6**, 30522, (2016).

16 van Kampen, M. *et al.* All-Optical Probe of Coherent Spin Waves. *Phys. Rev. Lett.* **88**, 227201, (2002).

17 Bigot, J. Y., Vomir, M., Andrade, L. H. F. & Beaurepaire, E. Ultrafast magnetization dynamics in ferromagnetic cobalt: The role of the anisotropy. *Chem. Phys.* **318**, 137-146, (2005).

18 Au, Y. *et al.* Direct Excitation of Propagating Spin Waves by Focused Ultrashort Optical Pulses. *Phys. Rev. Lett.* **110**, 097201, (2013).

19 Zhang, G. P., Latta, T., Babyak, Z., Bai, Y. H. & George, T. F. All-optical spin switching: A new frontier in femtomagnetism — A short review and a simple theory. *Mod. Phys. Lett. B* **30**, 16300052, (2016).

20 Greilich, A. *et al.* Mode locking of electron spin coherences in singly charged quantum dots. *Science* **313**, 341-345, (2006).
